# Protein S-glutathionlyation links energy metabolism to redox signaling in mitochondria

**DOI:** 10.1016/j.redox.2015.12.010

**Published:** 2015-12-31

**Authors:** Ryan J. Mailloux, Jason R. Treberg

**Affiliations:** a Memorial University of Newfoundland, Department of Biochemistry, St. John's, Newfoundland, Canada; b University of Manitoba, Department of Biological Sciences, Winnipeg, Manitoba, Canada; c Department of Human Nutritional Sciences, Winnipeg, Manitoba, Canada; d Centre on Aging, Winnipeg, Manitoba, Canada

**Keywords:** PGlu, protein S-glutathionylation, H_2_O_2_, hydrogen peroxide, GSH, glutathione, GSSG, glutathione disulfide, OXPHOS, oxidative phosphorylation, ROS, reactive oxygen species, Glutathione, Mitochondria, Redox signaling, Glutathionylation, Glutaredoxin, Hydrogen peroxide

## Abstract

At its core mitochondrial function relies on redox reactions. Electrons stripped from nutrients are used to form NADH and NADPH, electron carriers that are similar in structure but support different functions. NADH supports ATP production but also generates reactive oxygen species (ROS), superoxide ($O_{2}^{\cdot -}$) and hydrogen peroxide (H_2_O_2_). NADH-driven ROS production is counterbalanced by NADPH which maintains antioxidants in an active state. Mitochondria rely on a redox buffering network composed of reduced glutathione (GSH) and peroxiredoxins (Prx) to quench ROS generated by nutrient metabolism. As H_2_O_2_ is quenched, NADPH is expended to reactivate antioxidant networks and reset the redox environment. Thus, the mitochondrial redox environment is in a constant state of flux reflecting changes in nutrient and ROS metabolism. Changes in redox environment can modulate protein function through oxidation of protein cysteine thiols. Typically cysteine oxidation is considered to be mediated by H_2_O_2_ which oxidizes protein thiols (SH) forming sulfenic acid (SOH). However, problems begin to emerge when one critically evaluates the regulatory function of SOH. Indeed SOH formation is slow, non-specific, and once formed SOH reacts rapidly with a variety of molecules. By contrast, protein S-glutathionylation (PGlu) reactions involve the conjugation and removal of glutathione moieties from modifiable cysteine residues. PGlu reactions are driven by fluctuations in the availability of GSH and oxidized glutathione (GSSG) and thus should be exquisitely sensitive to changes ROS flux due to shifts in the glutathione pool in response to varying H_2_O_2_ availability. Here, we propose that energy metabolism-linked redox signals originating from mitochondria are mediated indirectly by H_2_O_2_ through the GSH redox buffering network in and outside mitochondria. This proposal is based on several observations that have shown that unlike other redox modifications PGlu reactions fulfill the requisite criteria to serve as an effective posttranslational modification that controls protein function.

## Highlights

• Mitochondria employ redox buffering networks to regulate bioenergetics.
• PGlu reactions link energy/nutrient metabolism to redox signaling.
• Other redox modifications lack regulatory features.

## Graphical abstract

**Figure:**
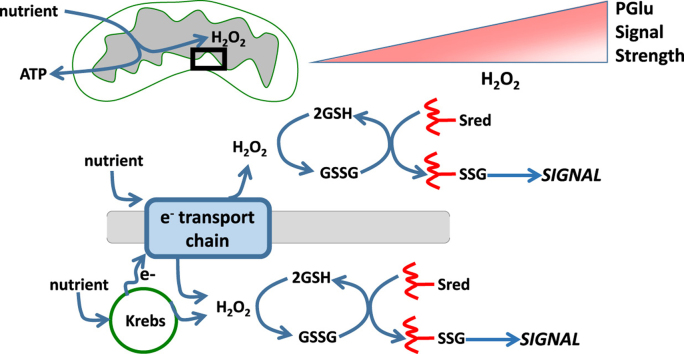

## 1 Introduction

Mitochondrial reactive oxygen species (ROS) production is being studied more than ever due its roles in physiology and disease. In particular high interest in mitochondrial ROS can be associated with the “free radical theory of aging”, which was put forth by Denham Harman in 1956, who posited that free radicals, chemical entities that contain one or more unpaired electrons, are responsible for aging as well as a range of disorders [1]. It follows that highly reactive free radicals generated by biological systems irreversibly damage macromolecular structures leading to tissue damage, development of various pathological conditions, and eventually death [2]. Mitochondria were eventually found to be a significant source of a cell's apparent free radical burden and these double membrane-bound organelles stood for many years falsely accused as the primary reason why aerobic organisms have a finite lifespan [2]. Now, it is appreciated that low grade mitochondrial ROS production has many cellular benefits [3]. However, ROS are still dangerous in large quantities, in excess to the cell's ‘normal’ levels, where they can overburden antioxidant systems leading to oxidative damage and cell death. But even excessively elevating levels of ROS serve as a signaling molecule activating cell proteins required to initiate apoptosis and necroptosis, the cell's “self-destruct” systems. With this, there is no doubt that unchecked ROS production is a constant danger which explains why antioxidant systems needed to develop in parallel with O_2_ utilizing pathways in the evolution of aerobic oxidative metabolism [4].

The term “ROS” is often utilized carelessly in many mitochondrial studies. There are a number of ROS that can be generated by cellular systems which differ considerably in reactivity and conditions under which they are produced. The chief ROS generated by mitochondria are superoxide ($O_{2}^{\cdot -}$) and hydrogen peroxide (H_2_O_2_), with the former considered by many to be the proximal ROS generated by mitochondria systems. Although $O_{2}^{\cdot -}$ is the proximal ROS generated in many cases, this molecule does not last long in solution due to the rapid activity of superoxide dismutase (MnSOD; matrix, Cu/ZnSOD; intermembrane space; IMS) [5]. Dismutation of 2$O_{2}^{\cdot -}$ generates one molecule of H_2_O_2_ and SOD, which is highly concentrated in mitochondria, dismutates $O_{2}^{\cdot -}$ at a rate of 1.8×10^9^ M^−1^s^−1^ indicating that following its production, $O_{2}^{\cdot -}$ is rapidly converted into H_2_O_2_ [6]. Production of both molecules is thermodynamically favorable with H_2_O_2_ occurring at far superior concentrations than $O_{2}^{\cdot -}$ [6], [7]. Production of $O_{2}^{\cdot -}$ and H_2_O_2_ also vary considerably in conjunction with the bioenergetic signature of mitochondria. During nutrient oxidation a fraction of the electrons can prematurely “spin-off” various electron donating sites to monovalently, or divalently, reduce O_2_ producing $O_{2}^{\cdot -}$ and/or H_2_O_2_ respectively. A myriad of factors converge on mitochondria to influence H_2_O_2_ formation and can act as key determinants for whether or not H_2_O_2_ will be utilized in signaling or cell death. This includes mitochondrial redox and bioenergetics poise, formation of supercomplexes or enzyme assembly, covalent modification, and factors that control the entry and exit of electron from sites of ROS production.

**Fig. 1:**
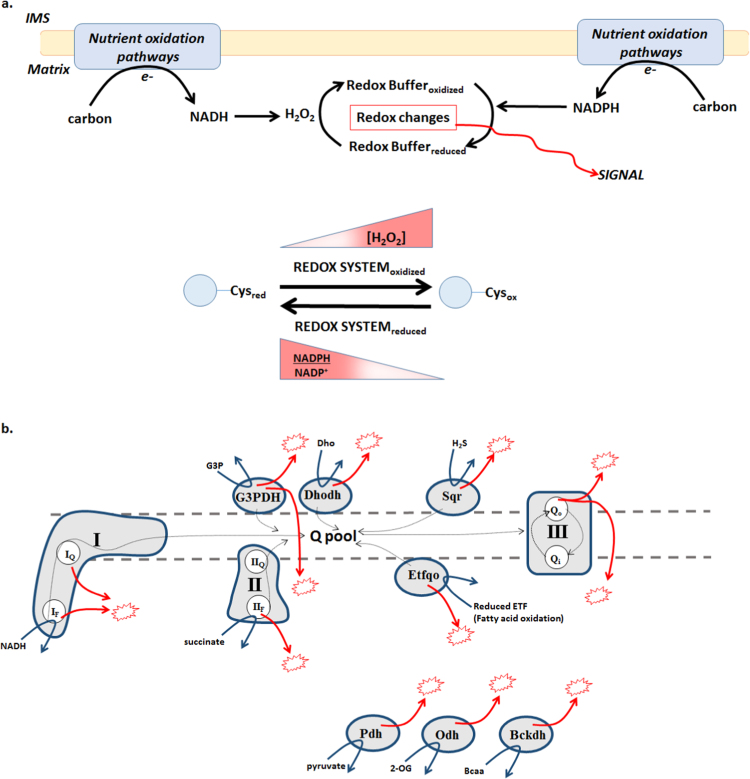
Mitochondrial redox flux and signaling. A. Nutrient catabolism and the liberation of electrons is coupled to the formation of NADH and NADPH which are utilized to support pro-oxidant and antioxidant activities in mitochondria. This results in spatiotemporal changes in mitochondrial redox buffering networks conveying signals that regulate protein function through cysteine switches. B. 10 potential sites for H_2_O_2_ production by mitochondria (represented by red star). Note the topology of H_2_O_2_ formation relative to the mitochondrial inner membrane. Sites include Complex I, Complex II (succinate dehydrogenase; Sdh), Complex III, 2-oxoglutarate dehydrogenase (Odh), pyruvate dehydrogenase (Pdh), branched-chain keto acid dehydrogenase (Bckdh), electron-transferring flavoprotein-ubiquinone oxidoreductase (Etfqo), *sn*-glycerol-3-phosphate dehydrogenase (G3PDH), dihydroorotate dehydrogenase (Dhodh), and sulfide quinone oxidoreductase (Sqr). FMN-Complex I; I_F_, quinone binding site Complex I; I_Q_, FAD-Complex II; II_F_, quinone binding site Complex II; II_Q_, quinone binding site outer leaflet Complex III; III_Qo_, quinone binding site inner leaflet Complex III; III_Qi_, 2-oxoglutarate; 2-OG, branched chain amino acid; Bcaa, dihydroorotate; Dho. (For interpretation of the references to color in this figure legend, the reader is referred to the web version of this article.)

It is now appreciated that cells contain an entire “*redoxome*” where protein cysteine thiols are utilized to sense changes in the surrounding redox environment [8]. Redox signals work in tandem with other signals to control various cellular processes like cell division, cell growth, metabolic output, wound healing, embryonic development, and cell death [9]. At its core, cellular redox signaling is initiated by nutrient metabolism since electrons stripped from nutrients during metabolism are utilized to generate NADH and drive oxidative phosphorylation (OXPHOS) but substrate oxidation also forms H_2_O_2_ (Fig. 1) [10]. Nutrients are also used to produce NADPH which is required to restore the mitochondrial redox buffering network after H_2_O_2_ has been scavenged by antioxidant systems like glutathione (Fig. 1). Control over H_2_O_2_ levels is governed by its production and removal. H_2_O_2_ levels are anticipated to be highest near sites of production decreasing in concentration as it diffuses away from its origin. This creates H_2_O_2_ gradients which would also generate redox buffering gradients with antioxidants being more oxidized near sites of H_2_O_2_ (Fig. 1). Hence, an “*ebb and flow*” exists with changes in mitochondrial redox environment since H_2_O_2_ formed by nutrient metabolism oxidizes local redox networks which are then recovered by NADPH. Such spatiotemporal fluctuations in cellular redox systems form a crucial part of a cell's “*Redox Code*”, a concept put forth recently by Jones and Sies, which stipulates that redox signature fluctuations are rooted in the state of nutrient metabolism and bioenergetics, production of NADH and NADPH, and the redox state of antioxidant systems [10].

Despite our widening knowledge on the importance of redox signals in modulating routine cellular functions, the molecular details surrounding how redox changes are communicated to proteins still remains enthusiastically debated. This may be due to the fact that cysteines can undergo a range of redox modifications which includes sulfenylation (SUF; formation of SOH by direct SH oxidation) and protein S-glutathionylation (PGlu) [11]. As discussed in detail by Shelton et al. [12], if a redox modification is to serve as a *bona fide* signal that controls proteins through posttranslational modification (PTM), it must fulfill certain criteria (Table 1) [12]. Shelton et al. discussed this in detail stating that redox signals should fulfill the same criteria as phosphorylation – *e.g.* must be specific, rapid, respond to physiological stimuli, must occur under physiological conditions (not just pathological), and must be reversible [12]. Further, Shelton et al. went on to describe how PGlu reactions fulfill all these criteria and thus likely serve as important PTM required to modulate protein function in response to changes in redox environment. After 10 years of research it is evident that PGlu reactions are required to reversibly regulate protein function in response to changes in redox environment. Moreover, it is now known that PGlu reactions play an important role in controlling mitochondrial functions ranging from metabolism to shape and protein import and loss of control over mitochondrial PGlu can lead to pathogenesis. Here, we provide an updated view on these concepts and argue that PGlu reactions form the link between mitochondrial oxidative metabolism and modulation of protein function by redox signaling.

## 2 Mitochondrial metabolism of $O_{2}^{\cdot -}$ and H_2_O_2_

### 2.1 Sources and link to nutrient oxidation

OXPHOS and mitochondrial “ROS” production are intimately linked to one another by the efficiency of mitochondrial electron transfer reactions. Meeting cellular ATP demand by OXPHOS is initiated when disparate macronutrients (carbohydrate, lipid and proteins) are converted to common intermediates which are oxidized by Krebs cycle enzymes yielding electrons predominantly captured in the cofactor NADH. NADH is oxidized at the level of Complex I, succinate at Complex II (succinate dehydrogenase; Sdh) while other ubiquinone oxidoreductase complexes (such as G3PDH, ETF-QOR) can also supply electrons to the mitochondrial quinone pool (Fig. 1b) [13]. Electrons travel through Complexes I and III reducing O_2_ to H_2_O at Complex IV [13].

A fraction of the electrons utilized in OXPHOS can prematurely exit the respiratory chain and react with O_2_ generating either $O_{2}^{\cdot -}$ which is then dismutated to H_2_O_2_ or in some cases enzyme complexes form H_2_O_2_ directly. Impairment of electron flow from nutrient oxidation to O_2_ reduction can amplify ROS production [14], [15], [16]. In addition, there is a non-Ohmic relationship between Δp and ROS production such that small changes in Δp can lead to a large differences in ROS formation [17]. Complex I and III of the electron transport chain are typically considered the chief sites for mitochondrial ROS formation but now it is well known that mitochondria can contain up to 10 sites, summarized in Fig. 1b. Important to the current synthesis, the 10 sites can be subdivided into two isopotential groups based on which electron donating group is involved in ROS production; NADH/NAD and QH_2_/Q groups [18], [19]. Moreover, many of the major ROS producing enzyme complexes also act as key entry sites for nutrient carbon oxidation by mitochondria, or alternative electron entry points in substrate oxidation, leading to a combined suite of factors that link nutrient oxidation and site-specific ROS formation (Table 2).

### 2.2 Topology and intertissue considerations

Classifying ROS generating sites in mitochondria based on what controls their capacity for producing $O_{2}^{\cdot -}$/H_2_O_2_ can be useful and has been discussed in detail elsewhere (Table 2) [18], [19]. However, the compartment(s) where ROS are released to, the topology, is also central to the cellular consequences of these ‘electron leaks’. Most enzyme complexes that produce ROS appear to release it into the matrix, whereas some sites (IIIQo and G3PDH) form ROS in the IMS or matrix. In regard to topology, H_2_O_2_ emitted into the IMS should have different signaling functions than H_2_O_2_ formed in the matrix. This is best illustrated in a recent study where Bleier et al. found that H_2_O_2_ produced in the matrix has different signaling targets than when it is generated in the IMS [20]. In fact, the authors called it “*generator specific targets*” for mitochondrial redox signaling in reference to the importance in topology in influencing redox signaling [20]. Another consideration is that mitochondria generate variable amounts of H_2_O_2_ in the presence of different substrates in a tissue-dependent manner. For example succinate is an important substrate for the production of H_2_O_2_ in brain, heart, kidney, and skeletal muscle whereas fatty acids may produce substantial amounts in liver [21]. By contrast skeletal muscle mitochondria can have high rates of production when metabolizing succinate or glyceraldehyde-3-phosphate in comparison to palmitoyl-carnitine or glutamate/malate [22]. Tissue-specific variation in the hierarchy of ROS producing pathways likely reflects differences in the intermediary metabolism across tissue types, and the concomitant differential levels of expression for enzymes of the nutrient oxidation pathways.

### 2.3 The fate of H_2_O_2_

**Fig. 2:**
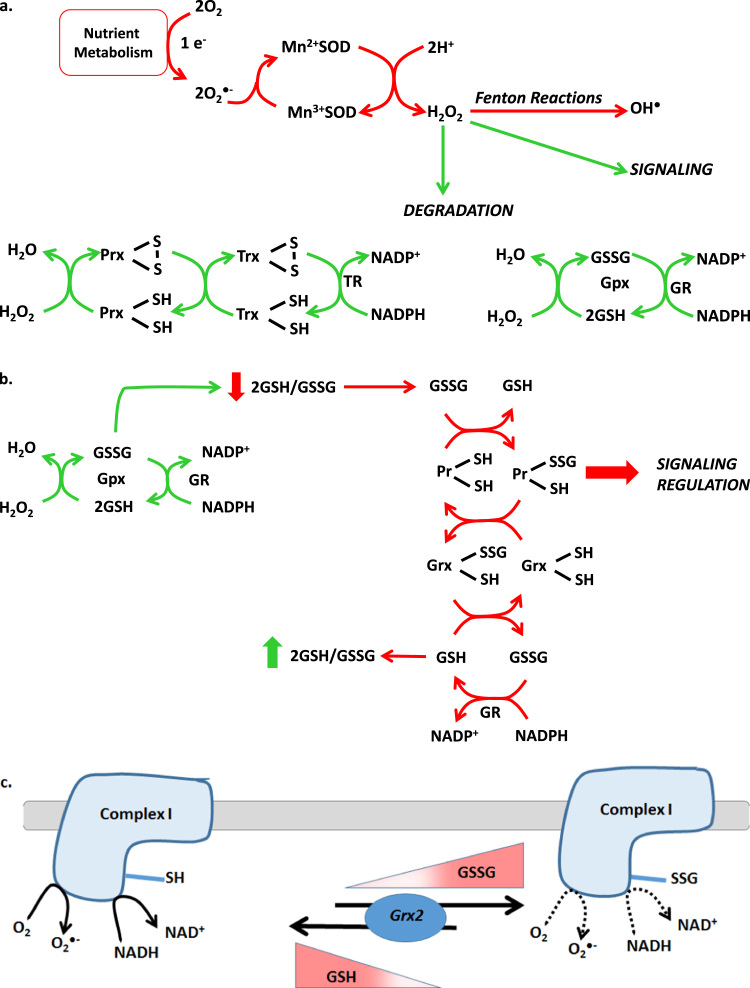
Protein S-glutathionylation in redox signaling A. Mitochondrial redox buffering systems B. S-glutathionylation and the formation of reversible protein glutathione mixed disulfides as a potential mechanism for H_2_O_2_-mediated signaling inside and outside of mitochondria. C. Control of Complex I by reversible protein S-glutathionylation reactions.

Following its production, either directly or from superoxide dismutation, H_2_O_2_ is either metabolized by antioxidant systems or used in signaling [23], [24]. The latter trait is associated with several factors including its capacity to diffuse through membranes, ability to react with thiols, and its longer half-life in solution which is influenced by its rate of degradation. Although H_2_O_2_ is used in cell signaling, levels of this molecule must remain under strict control since high concentrations can induce oxidative damage through Fenton and Haber–Weiss reactions [25], [26]. H_2_O_2_ is quenched by two main redox systems; the glutathione and peroxiredoxin (Prx) systems (Fig. 2a). Both systems have been reviewed extensively [7], [27], [28], [29]. In brevity, the glutathione system utilizes glutathione peroxidase (GPx) in the presence of two reduced glutathione (GSH) molecules to sequester H_2_O_2_ generating oxidized glutathione (GSSG) and water [30]. GSH is then recovered by glutathione reductase in the presence of NADPH (Fig. 2a). On the other hand Prx quenches H_2_O_2_ using a highly reactive peroxidatic cysteine residue which is resolved via disulfide bridge formation followed by reactivation via thioredoxin (Trx), thioredoxin reductase (TR), and NADPH (Fig. 2a) [31]. The reductive power stored in NADPH is required to drive both peroxidase systems. NADPH is also produced as a consequence of nutrient metabolism in mitochondria and throughout the cell where it serves as an important counterpart to the NADH system since NADPH supports both anabolic and antioxidant systems [10].

## 3 Protein S-glutathionylation (PGlu) as a signaling mechanism

### 3.1 Sulfenylation vs. PGlu as likely mechanism

Sulfenylation and formation of PGlu are the two primary candidates for linking H_2_O_2_ to redox signaling cascades. There are a few proteins that have been documented to undergo SUF in mitochondria, however, when criteria in Table 1 are considered it seems unlikely that H_2_O_2_ mediates redox signals in mitochondria. First, H_2_O_2_ weakly reacts with a majority of protein cysteine thiols [32]. Prx and GPx, which combined are highly concentrated in mitochondria, quench H_2_O_2_ at rates of ~10^7^ M^−1^ s^−1^ which would suggest that H_2_O_2_ is being cleared before it can serve as an effective signaling molecule [32]. Indeed, when isolated mitochondria are incubated with many respiratory substrates there is a failure to accumulate H_2_O_2_ in the medium because the matrix consumers destroy the vast majority of the total H_2_O_2_ produced [33]. It has also been reported that SOH groups are highly unstable reacting with various other groups, like GSH, at rates>10^5^ M^−1^ s^−1^ [34]. This means that SUF formation lacks specificity since SOH groups are highly unstable and able to react with a variety of electrophiles. In addition, although SUF reaction products, like formation of SO_2_H, disulfide bonds such as PGlu, and intra- or intermolecular disulfide bridges between protein cysteine thiols, are reversed by sulfiredoxins, glutaredoxins, and thioredoxins, it would seem that reversal of these modifications serves as a defense mechanism to protect enzymes from irreversible deactivation [35]. This makes it very difficult to reconcile how H_2_O_2_ operates with adequate specificity, sensitivity, and reversibility to serve as regulatory device.

When weighed against the criteria in Table 1, it would seem that H_2_O_2_ does not conduct redox signals directly to proteins. However, this does not mean that H_2_O_2_ cannot manipulate redox signals indirectly by modulating redox buffering systems. To this end, PGlu are a strong candidate for mediating mitochondrial nutrient-driven redox signals since (1) this redox reaction meets all criteria in (Table 1, Table 2) changes in GSH/GSSG, which are driving force behind PGlu reactions, are directly influenced by H_2_O_2_ flux (Fig. 2b) [36], [37]. PGlu reactions are enzymatically regulated by glutaredoxins (Grx), in particular Grx1 which is found in the cytosol and IMS and Grx2 in the matrix of mitochondria (Fig. 2b) [38]. In addition both enzymes occur at high concentrations with Grx2 found in low μM concentrations in mitochondria [38]. Also, S-glutathionylation can proceed spontaneously if GSSG is high enough but it has also been reported that protein S-glutathionylation can proceed via the formation of a protein thiyl radical which subsequently reacts with deprotonated GSH [39]. Spontaneous S-glutathionylation by simple thiol disulfide exchange between GSSG and a protein SH group was originally only thought to proceed during oxidative stress (*e.g.* when GSSG levels reach 1 mM surpassing GSH) [40]. However, it is now appreciated that local regions of low GSH/GSSG (e.g. high GSSG) are generated naturally due to changes in H_2_O_2_ flux [28]. This is highly relevant to mitochondria since the inner folding of the mitochondrial inner membrane (MIM) can microcompartmentalize GSH/GSSG gradients [28]. Although spontaneous PGlu can occur, it has been well documented that Grx1 and Grx2 can also catalyze the S-glutathionylation of protein targets which depends on the redox state of GSH/GSSG or the formation of thiyl radicals on GSH [40], [41]. Specifically, Grx2 has been found to catalyze the S-glutathionylation of Complex I when GSH/GSSG is low and H_2_O_2_ production by mitochondria is high (Fig. 2c) [41], [42]. Likewise when GSH/GSSG levels have been restored to ~50 by GR and NADPH, Grx2 deglutathionylates Complex I (Fig. 2c) [41], [42]. Thus, it is probable that H_2_O_2_ serves as a secondary messenger which is required to prime redox signaling pathways through PGlu reactions (Fig. 2b).

### 3.2 Linking Grx2 regulation and mitochondrial ROS formation

The potential linkage between mitochondrial ROS formation and Grx-mediated regulation of protein modifications becomes intrinsic because Grx2 activity is directly regulated by ROS [43], [44]. Grx2 is maintained as a catalytically inactive dimer through coordination of a 2Fe–2S cluster in conjunction with two GSH molecules [45]. A burst of $O_{2}^{\cdot -}$ production leads to the disassembly of the 2Fe–2S cluster and the release of two enzymatically active Grx2 monomers and 2 GSH molecules illustrating the intimate link between ROS flux in mitochondria and PGlu reactions further [46].

### 3.3 PGlu as a post-translational modification: parallel with phosphorylation

Since Grx2 is activated by ROS it would indicate that there is a second layer of regulation for PGlu reactions, much like phosphorylation cascades where kinases are activated and deactivated by physiological stimuli. However, in the case of Grx2 regulation is mediated by changes in electron flow and redox state which are coupled to assembly and deassembly of Fe–S clusters. Much like phosphorylation motifs proteins also display PGlu motifs which are usually characterized by a cysteine residue flanked by several positively charged amino acids [12], [47]. Finally, along with containing high levels of the requisite enzymes, the physical properties of mitochondria imbue them with the capacity to drive PGlu reactions which has been reviewed extensively in [35], [48]. Briefly, mitochondria contain a high concentration of solvent accessible thiols including high amounts of glutathione (GSH~5 mM and GSSG~0.1 mM setting GSH/GSSG at ~50) with GSH being the predominant redox buffer in mitochondria [49]. The proton disequilibrium set to produce a high Δp results in a mildly alkaline matrix environment, which lowers the fraction of protonated cysteine thiols, and is an important linkage between nutrient oxidation and H_2_O_2_ formation [35]. When viewed in this manner, it is easy to reconcile how PGlu serves as the link between changes in electron flow and redox regulation of proteins.

## 4 PGlu reactions link metabolism to redox regulation of proteins

Mitochondria harbor a number of S-glutathionylation targets which control mitochondrial metabolism and function in response to fluctuations in redox environment [29], [50]. Krebs cycle enzymes, OXPHOS complexes, solute anion carriers, antioxidant enzymes, and proteins involved in controlling mitochondrial shape, protein import, and induction of apoptosis are all targets for regulation by PGlu [35], [51]. For Krebs cycle enzymes and OXPHOS complexes, PGlu reactions due to an increase in H_2_O_2_ typically result in decreased enzyme activity [29]. This is generally attributed to an increase in GSSG levels which results in spontaneous PGlu of protein targets in aconitase (Acn), NADP-dependent Idh, Ogdh, Complex I, Complex III, and Complex V (reviewed in [29,37]). However, not all mitochondrial proteins are negatively-modulated by PGlu. It has been documented that some proteins in mitochondria are basally S-glutathionylated in normal mitochondria which is required to maintain their function. For example, Sdh has been reported to be maintained in an S-glutathionylated state in normally functioning cardiac mitochondria [52]. Ischemia-reperfusion injury to the myocardium results in deglutathionylation of the Sdha subunit which decreases Complex II activity while simultaneously amplifying ROS production [52]. Adenine nucleotide translocator (ANT), which is required for ATP/ADP exchange, is also basally S-glutathionylated [53]. Decreased PGlu of ANT is associated with mitochondrial permeability transition pore (MPTP) opening and apoptosis in neurological tissue [53]. Similarly cyclophilin D is S-glutathionylated which prevents induction of mitoptosis [54].

To complicate matters further GSH is also able to S-glutathionylate target proteins. This is dependent on either protein thiyl radical formation (e.g. modification of a cysteine by another radical such as $O_{2}^{\cdot -}$) which reacts spontaneously with GSH or thiyl radical formation on GSH which, through Grx, can PGlu a target protein. $O_{2}^{\cdot -}$ has been found to drive the formation of thiyl radicals in mitochondria but other factors can also influence GSH-mediated PGlu including deprotonation of GSH or formation of glutathionyl radicals (GS^·^) [40], [55]. An excellent example of this is Complex I, which is a major site for PGlu-mediated control in several tissues (reviewed extensively in [37]). A burst in mitochondrial $O_{2}^{\cdot -}$ production leads to thiyl radical formation on Complex I which results in its conjugation to GSH [39], [56]. What is more is that this reaction proceeds even when GSH/GSSG is high [39]. Grx2 was only identified a little over a decade ago meaning that our understanding of how PGlu reactions are modulated and mediated is still in its infancy [57], [58]. However, after a decade of research the cumulative evidence indicates that PGlu reactions may play a central role in modulating mitochondrial function in response to changes in redox environment.

### 4.1 PGlu in modulating mitochondrial nutrient metabolism

**Fig. 3:**
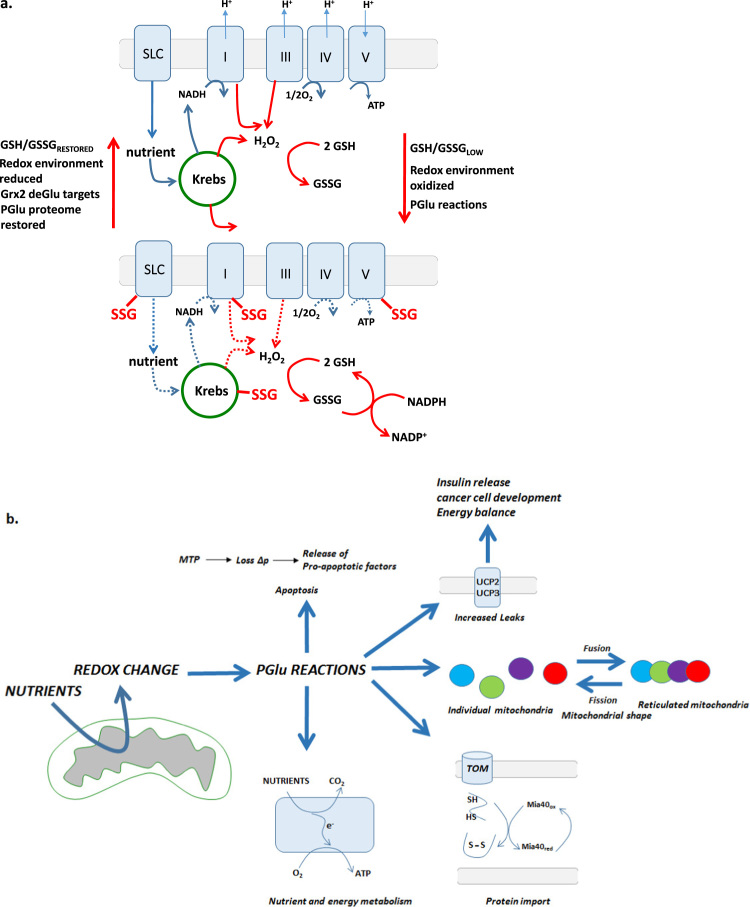
Protein S-glutathionylation reactions links changes in metabolism to control over proteins by redox signaling. A. Changes in nutrient metabolism in mitochondria either through direct modulation of nutrient uptake or oxidation alters NADH and H_2_O_2_ production. This results in spatiotemporal fluctuations in the redox state of mitochondrial redox buffering systems, principally GSH, which is then utilized to signal the state of the redox environment to proteins involved in various processes throughout mitochondria. Signaling is mediated by the direct covalent modification of various proteins which control mitochondrial shape, protein import, and other cellular processes like cell division, mechanical movement in muscle cells, vascular, cardiac, and neurological development and likely many others. Protein S-glutathionylation also feeds back on mitochondria to control nutrient uptake, metabolism, electron flux, and ROS production. This ultimately alters the bioenergetic signature of mitochondria leading to an overall slowing of oxidation reactions limiting the supply of electrons for NADH and H_2_O_2_ formation. Due to the decrease in ROS formation and the provision of NADPH, mitochondrial redox buffering systems are recovered restoring the redox environment which ultimately drives reversal of protein S-glutathionylation restoring nutrient oxidation reactions. B. Effects of mitochondrial PGlu reactions and changes in redox environment on cell physiology.

Metabolic organization serves as the foundation of the Redox Code [10]. Changes in carbon flux and electron supply during nutrient oxidation directly influence the state of the redox environment through shifts in NADH, NADPH, and H_2_O_2_ production and changes in the mitochondrial redox buffering network. Through these interacting metabolite pools redox signaling provides a direct link between mitochondrial metabolism and control over protein function [10]. As described above, it is not clear if H_2_O_2_ mediates nutrient driven redox signals directly since it fails to fulfill some of the criteria required for a covalent modification to serve as an effective means to control proteins [12]. Rather protein S-glutathionylation is a strong candidate for linking changes in metabolism to modulation of protein function [10]. Two key examples highlighting how PGlu reactions serve as this link are (1) a function in directly controlling nutrient supply and carbon flux and (2) the role in modulating mitochondrial ROS formation. In regard to carbon flow, as discussed above and reviewed elsewhere, PGlu reactions converge on the Krebs cycle to modulate several enzymes [27], [37], [59]. PGlu reactions alter Krebs cycle flux resulting in significant increases or decreases in the concentration of different Krebs cycle intermediates which may be associated either with the direct inhibition of different enzymes or changes in anaplerotic or cataplerotic flux [42], [60]. PGlu may also control nutrient delivery into mitochondria or carbon entry into oxidative pathways at three important points; (i) carnitine/acyl-carnitine translocase (CACT); (ii) Ogdh, a major site of carbon entry into the Krebs cycle from amino acid catabolism/oxidation and (iii) Pdh, the major control site for oxidation of carbohydrate-derived carbon, which would not only control mitochondrial bioenergetics at the level of substrate supply but could also modulate ROS production (Fig. 3).

Mitochondrial oxidation of long-chain fatty acids requires the mitochondrial import of long-chain fatty acyl carnitine esters; CACT is an antiporter embedded in the MIM that exchanges acyl-carnitines for l-carnitine from the matrix [61]. However, the capacity for exchange by CACT is inhibited by formation of PGlu when GSH/GSSG is low [62], which will impede the oxidation of long-chain fatty acids. Supporting a role for PGlu formation inhibiting lipid oxidation, it has been documented that loss of control over PGlu reactions in cardiac mitochondria results in a substantial decrease in fatty acid-supported OXPHOS [42]. In addition, deregulated PGlu reactions have been linked to excessive fat accumulation in adipocytes and the development of obesity [63].

Ogdh and Pdh are multienzyme complexes that utilize various cofactors and both are tightly regulated by a number of factors including phosphorylation and various allosteric activators and inhibitors. While major entry points for amino acid or carbohydrate derived carbon into the Krebs cycle, Ogdh and Pdh respectively, are also highly sensitive to redox regulation meaning that their capacity to commit carbon to further oxidation is controlled by redox signaling. Fewer studies have focused on the function of PGlu in modulating Pdh however recent evidence does indicate that it is controlled by changes in mitochondrial redox buffering systems [64], [65]. For Ogdh PGlu is required to protect the enzyme complex from irreversible deactivation by ROS which can be reversed by Grx proteins [66]. However, considering that PGlu lowers Ogdh activity, S-glutathionylation may also be required to enhance amino acid biosynthesis through diversion of 2-oxoglutarate towards glutamate production (Fig. 3). Taken together, PGlu reactions are effective mediators of redox signals, linking changes in mitochondrial redox buffering capacity to modulation of nutrient delivery and oxidation in mitochondria.

### 4.2 ROS production and other mitochondrial functions

The fact that redox signals are influenced by spatiotemporal changes in H_2_O_2_ demands that these signals also feedback to negatively regulate ROS formation. By doing so the redox signal can be shut off by limiting ROS formation and ensuring NADPH can be used to restore redox buffering environments. This is indeed the case for PGlu reactions which have been shown to limit mitochondrial ROS production (Fig. 3). Most studies have focused on the modulation of Complex I which harbors 3 S-glutathionylation sites (reviewed in [37]). Reversible PGlu of Ndusf1 subunit lowers Complex I activity by physically blocking the NADH oxidation site [41], [67]. This has the added benefit of lowering ROS formation by Complex I and the rest of the electron transport chain until GSH/GSSG is restored by NADPH and GR resulting in the Grx2-mediated deglutathionylation of Ndusf1 (Fig. 3) [41]. It has also been documented that deregulated PGlu reactions in mitochondria negatively affect the capacity of mitochondria to support Complex I-mediated OXPHOS which correlates with amplified mitochondrial ROS production, development of heart disease, cataracts, and neurological disorders [42], [68], [69]. When Complex I is inactivated by PGlu over prolonged periods, it is likely ROS production from the other sites in mitochondria will increase (Table 2 and Fig. 1b) [37]. Since PGlu modulates ROS production by Complex I, it is also important to consider if PGlu also controls production from the other ROS-emitting sites in mitochondria (Table 2). As discussed above, PGlu controls ROS formation from Sdh which has been shown to serve as a potent $O_{2}^{\cdot -}$/H_2_O_2_ generating site [70]. Ogdh and Pdh are also significant sources of ROS and targeted for regulation by redox signaling. It is unknown if PGlu modulates ROS emission from either enzyme complex however; considering that PGlu lowers Ogdh activity and depletion of mitochondrial glutathione amplifies ROS formation by Pdh then it is reasonable to propose that PGlu controls ROS formation by either enzyme [64], [66]. Whether or not PGlu reactions can control ROS production or enzyme activity of the several other ROS forming mitochondrial complexes remains unexplored.

Since Δp and mitochondrial ROS formation are intrinsically linked, further evidence comes from the finding that PGlu reactions are required for the reversible regulation of proton leaks in skeletal muscle, thymus, cancer cells, and pancreatic islets. This is achieved through the ROS-sensitive reversible PGlu of uncoupling protein (UCP)-2, which is more ubiquitously expressed, and UCP3, which is expressed mostly in skeletal muscle [29], [71]. Through a series of publications it was found that UCP2 and UCP3 are maintained in an S-glutathionylated state when Δp is low [72]. A rise in Δp and a subsequent increase in mitochondrial H_2_O_2_ formation results in the deglutathionylation of UCP2 and UCP3 activating proton leaks [72], [73]. This effectively lowers mitochondrial ROS formation. The rise in ROS due to an increase in membrane potential also activates Grx2 which is required to drive reglutathionylation [60].

### 4.3 Physiological implications and evidence

The physiological importance of the PGlu-dependent regulatory mechanism has been discussed in some detail in several reviews [28], [29], [35], [59], [74]. In brevity, the physiological effects range from modulating insulin release from β-cells to serving as a target for sensitization of cancer cells overexpressing UCP2 and UCP3 towards chemotherapy (overexpression of either is used to fend ROS and chemical PGlu deactivates either protein enhancing the effectiveness of chemotherapy). Intriguingly Grx2−/− mice also show signs of decreased adiposity and a significant decrease in overall fat mass and skeletal muscle triglyceride levels [60]. This was associated with the chronic activation of proton leaks through UCP3 in skeletal muscle pointing the possibility that PGlu reactions play a fundamental role in modulating overall energy balance in the body (Fig. 3) [60]. Emphasizing the importance of topology and compartmentalization, the potential role for PGlu regulation in mitochondria is not limited to the matrix. Mitochondrial PGlu reactions are required to regulate fission and fusion events where a more oxidized GSH pool enhances mitochondrial fusion and the genesis of a hyper-reticulated mitochondrial network [75]. Further, changes in redox buffering capacity in the IMS has also been linked to the control of mitochondrial protein import. Recent work has found that the Mia40-Erv1p pathway, which is required for the oxidative folding of proteins prior to entry into the matrix, is also targeted for S-glutathionylation and Grx action (Fig. 3) [51].

Much still remains to be elucidated in regard to the function of PGlu reactions as a feedback regulatory mechanism for mitochondrial ROS formation. Nonetheless, results collected so far indicate that PGlu reactions in mitochondria are required to control nutrient metabolism and ROS formation in response to fluctuations in the mitochondrial redox buffering system.

## 5 Summary and perspectives

Mitochondria are central to energy metabolism, generating ATP from the oxidation of nutrients and the flow of electrons and protons. To safeguard itself from the imperfections associated with electron transfer reactions, mitochondrial redox buffering networks are used to quench ROS. The emergence of more sensitive methods of measurement has shown that redox buffering networks play a central role in intra- and extra-mitochondrial signaling, linking shifts in nutrient metabolism and flux through important redox mediating metabolites to the modulation of proteins. These signals have a number of functions ranging from modulation of nutrient metabolism and ROS production to regulating protein import, mitochondrial shape, apoptosis and many others. It is also clear that redox signals originating from mitochondria may also play a fundamental role in modulating cellular functions which is evidenced by the pathological effects associated with disabling these key redox signaling systems. Cysteine switches can be subjected to a broad range of modifications but only PGlu reactions meet the requisite criteria to regulate protein function. Protein S-glutathionylation has all the characteristics of a classic covalent modification typically utilized in reversible regulation of proteins. PGlu reactions are also highly sensitive to changes in redox environment which is associated with the role of GSH in serving as the major redox buffer in any cellular environment. Taken together, PGlu reactions combined with the substrate-dependence and topology of mitochondrial ROS formed represent a potential functional link integrating nutrient metabolism, spatiotemporal fluctuations in redox environment, and control over mitochondrial function and cellular physiology.

## Figures and Tables

**Table 1:** Criteria for covalent modifications to serve as a regulatory mechanism. Chart lists the different criteria that must be met for a posttranslational modification to serve as a regulatory mechanism. Criteria were generated based the function of binary switches, like phosphorylation, in the control of protein function through alterations in structure. The Table was adapted from [12].

**Criteria**	**Sulfenylation**	**Protein S-glutathionylation**
Change function of protein	Yes	Yes
Occurs in response to physiological stimuli	Yes	Yes
Modification is rapid and enzymatically mediated	Kinetically slow and not enzymatically mediated	Yes
Modification is controlled and site specific	Unknown	Yes
Modification is stable and does not lead to unwanted side reactions	No	Yes
Modification is reversible	Yes	Yes

**Table 2:** Major sites of mitochondrial reactive oxygen species (ROS) production and energetic linkages or major nutrient oxidation pathways associated to the control of ROS production from each site^a^. Note that Dhodh and Sqr have been excluded from this table since supraphysiological concentrations of dihydroorotate are required to generate ROS by Dhodh and ROS genesis by Sqr is poorly characterized. Electron transport chain (ETC).

**Site**	**Energetic control/Linkage to ROS production**	**Nutrient/metabolic pathway(s)**
Complex I	NADH/NAD^+^, QH_2_/Q, Δp^b^ and NAD-pool size	Krebs cycle, OXPHOS/ETC
Complex II	QH_2_/Q, and [substrate]^c^	Krebs cycle
Complex III	QH_2_/Q, Δp	OXPHOS/ETC
Pdh	NADH/NAD^+^, [pyruvate]	Carbohydrate oxidation
Odh	NADH/NAD^+^, [oxoglutarate]	Krebs cycle, amino acid oxidation/transamination
Bckdh	NADH/NAD^+^, [branched chain amino acids]	Amino acid oxidation
G3PDH	QH_2_/Q, [G_3_P]	Carbohydrate oxidation, triglyceride catabolism (to lesser extent)
Etfqo	QH_2_/Q, reduced ETF	Beta-oxidation of lipids, branched chain amino acid oxidation

a This list focuses on aspects relevent to the current paper and is not intended to be truly comprehensive.

b In general there is a positive relationship between increasing Δp and ROS production but it is important to appreciate that for all sites linked to NADH/NAD^+^ or QH_2_/Q there will be an indirect linkage with Δp because of the feedback effect of Δp on electron flux; however, here we indicate that for some sites there will also be a direct influence due to the coupling of electron flux through the complex and generation of the Δp.

c High levels of 4-carbon intermediates, including succinate, inhibits ROS formation from this site.
